# Prediction of the Risk Factors of Knee Injury During Drop-Jump Landing With Core-related Measurements in Amature Basketball Players

**DOI:** 10.3389/fbioe.2021.738311

**Published:** 2021-09-22

**Authors:** Liang Guo, Jing Zhang, Ying Wu, Li Li

**Affiliations:** ^1^ School of Physical Education & Sports Science, South China Normal University, Guangzhou, China; ^2^ Department of Obstetrics, Guangdong Maternal and Child Health Care Hospital, Guangzhou, China; ^3^ School of Physical Education and Training, Shanghai University of Sport, Shanghai, China; ^4^ Georgia Southern University, Department of Health Science and Kinesiology, Statesboro, GA, United States

**Keywords:** core stability, strength, motor control, knee injury, drop jump

## Abstract

**Purpose:** To evaluate the relationship between specific aspects of core stability and knee injury risk factors during drop-jump (DJ) landing.

**Methods:** Eighteen college-aged male amateur basketball players participated in the project. Kinetic and kinematic data for DJ tasks were collected with force plates and infrared cameras. Raw data were processed to calculate knee joint angles and joint moments during DJ landing. Different components of core stability were represented by the sit-ups in 20 s (SU), trunk extensor endurance, trunk flexion and extension range of motion, dominant extremity single-leg stance time (DLS), and dominant extremity single-leg hop distance, respectively.

**Methods:** Correlation and regression were used to determine the relationship between jumping-related biomechanical parameters and core stability components.

**Results:** SU shared significant variance with the peak moment of knee extension (PMKE, *p* < 0.05), the peak moment of knee abduction (PMKA, *p* < 0.05), and the angle of knee internal rotation at initial contact (AKRI, *p* < 0.05). DLS shared significant variance with the angular motion of knee internal rotation (AMKR, *p* < 0.05) and the AKRI (*p* < 0.01). SU and DLS together could explain 52% of the variance observed in the AKRI, and the result was significant.

**Conclusion:** Core stability’s strength and motor control aspects played an essential role in preventing knee injury during DJ landing. An integrative training program addressing core strength and motor control could be considered for coaches and athletes to prevent knee injury through core training and conditioning.

## Introduction

Landing is a frequent movement in basketball, volleyball, etc., which need to jump frequently (Dufek, 1991; Vander Does et al., 2016). On average, a basketball player performs 70 jumps in a game, and volleyball players jump approximately 60 times during 1 h of gameplay (Vander Does et al., 2016). The lower-extremity usually needs to bear ground reactional impact about 3.5–7 times of whole body weight in every take-off and landing (Fu et al., 2013). The high and sudden ground reaction forces produced by landings translate into large external torques at the knee that can easily lead to soft tissues injury, especially for ACL injury (Bates et al., 2013; Padua et al., 2015). 45% of the knee injuries of basketball and volleyball players occur when they land after take-off (Vander Does et al., 2016). Knee injury often affects athletic performance in competitive areas and adversely affects careers (Xu et al., 2020).

Previous research has studied the relationship between knee biomechanics during landing and knee injuries (Hewett et al., 2005; Della Villa et al., 2020). The research of Hewett et al. (2005) demonstrated that knee abduction angles and moments were the primary predictors of ACL injury risk. Increased abduction angles and moments on the knee can increase anterior tibial translation and loads on the ACL several-fold and lead to injury (Della Villa et al., 2020). Everard et al. (2019) and Dai et al. (2012) emphasized the combined effects of less knee flexion and more significant knee internal rotation and knee abduction on a greater risk of knee injury in sports. Incorrect positioning of the knee during landing could generate an additional load on the knee and lead to injury (Nguyen et al., 2015; Della Villa et al., 2020).

Core stability is vital in keeping the lower-extremity correctly positioned and decreases the risk of knee injuries during landing (Leetun et al., 2004; Myer et al., 2008; Araujo et al., 2015). Zazulak et al. (2007a) conducted a follow-up study on the relationship between core stability and knee injury in 277 college athletes. They reported that athletes who sustained knee injuries showed significantly weak trunk stability in the preseason test. An inadequate core may compromise the dynamic stability of the knee and result in an increased abduction moment, which may increase strain on the knee ligaments and lead to injury (Zazulak et al., 2007a). Tsai et al. (2019) observed that 6 weeks of core training could significantly reduce the trunk flexion angle and the maximum knee internal rotation angle of young volleyball players during drop-jump (DJ) landing. Correct knee positioning due to stronger core stability could reduce the overturning and rotating torque of the knee when landing and effectively reduce the risk of knee injury (Myer et al., 2008; Bagherian, 2018).

Therefore, core stability plays a crucial role in decreasing knee loading and preventing knee injury during landing. However, integrative training, including a variety of core stabilities, has been employed in previous studies to examine the relationship between core stability and knee injury risk (DiStefano et al., 2016; Tsai et al., 2019). More than two aspects were chosen: strength, endurance, flexibility, motor control, and function of core stability in the integrative training. Different aspects of core stability play different roles in preventing knee injury. It is difficult to determine which training elements are responsible for preventing knee injury during landing (Parsons et al., 2017; Guo et al., 2020). Understanding the benefits of different types of core training for reducing knee injury risk is essential to increase core training efficiency and prevent knee injury.

The specific aspects of core stability influencing knee injury should be examined and used to prevent knee injury. De Blaiser and colleagues (De Blaiser et al., 2019) identified the lack of core strength and endurance as risk factors for lower extremity overuse injuries when examining core stability and its relationship with overuse injuries in the lower extremity. However, in addition to core strength and endurance, flexibility, motor control, and functionality should also be examined to evaluate core stability comprehensively; see Table 1 for more details (Waldhelm, 2012; Guo et al., 2018a). Guo et al. (2018a) and Guo et al. (2020) examined the relationship between the five components of core stability and countermovement jump (CMJ) performance. They reported that the strength and function aspects of core stability were specific predictors of CMJ height. The relationship between the five components of core stability and the knee injury risk is still unknown. Better understanding this relationship could influence the efficiency of core training to prevent knee injury. In the present study, we tested dynamic trunk strength, trunk extensor endurance, trunk flexion and extension range of motion, single-leg stance time, and single-leg hop distance (Waldhelm, 2012; Guo et al., 2018a; Guo et al., 2019), representing the five components of core stability. These five variables represent the core’s strength, endurance, flexibility, motor control, and function, respectively. The purpose of the project was to investigate the relationship between different components of core stability and knee injury risk during DJ landing. We hypothesized that knee abduction moment and internal rotation were correlated with core-related measurements, but different components of core stability could have different contributions.

**TABLE 1:** The five components of core stability (Waldhelm, 2012; Guo et al., 2019)

Components	Strength	Endurance	Flexibility	Motor control	Function
Definition	The ability of the neuromuscular system of the core to overcome or resist external resistance.	The ability of the neuromuscular system of the core to maintain a specific intensity load or movement quality for a certain period of time.	The range of motion of the core, which is based on the elasticity and extension of its ligaments, tendons, muscles, skin and other tissues.	The ability of the core to contribute to keep a posture stable or a movement in intended trajectory.	The ability of the core to contribute to the function movement.
Related measurements	Trunk flexion; Trunk extension; Right hip extension; Left hip extension; Right abduction; Left abduction; Right hip ER; Left hip ER	Trunk flexion; Trunk extension; Right Side Bridge; Left Side Bridge	Sit and Reach; Trunk flexion; Trunk extension; Right trunk rotation; Left trunk rotation; Right hip extension; Left hip extension; Right hip IR; Left hip IR; Right hip ER; Left hip ER	Right SLB vision; Left SLB vision; Right SLB blindfold; Left SLB blindfold; Right hip reposition; Left hip reposition	Squat; Right hop distance; Left hop distance; Right hop timed; Left hop timed

ER, External Rotation; IR, Internal Rotation; SLB, Single leg balance test.

## Materials and Methods

### Participants

A convenient sample of eighteen college-aged amateur male basketball players was recruited using flyers and in-class announcements from a university. Regular basketball practice (more than 3 times per week for longer than 1 h each time) in the past 2 years was required to participate. Anyone with lower back or lower-extremity injuries or disease within the previous year was excluded from participation.

### Testing Procedure

Age, body mass, and height were recorded. Each participant was assigned an ID to protect the participant’s privacy. Before testing, each participant was asked to perform a 5 min warm-up following a “quick warm-up cardio workout” video. The warm-up exercise included the boxer shuffle, overhead reaching, stretching, high knee marching, torso twists, toe-touch kicks, full torso circles, lateral step toe touches, squats, jumping jacks, and high knees. There were two testing sessions, with a 30 min rest in between, for all participants. During the testing sessions, a DJ test and a core-related measurements test were performed. The experimenter demonstrated each test movement and participants practiced as many times as necessary before the actual test, typically two or three times. The DJ test was performed before the core-related measurements test to avoid potential fatigue effects. After completing the tests, each participant was asked to cool down as instructed in a video to reduce potential muscle soreness. The cool-down exercise included torso twists, rocking side kickers, rocking butt kickers and ventral pulls, arm crossover swings, quadriceps stretching, hamstring stretching, rocking inside thigh stretching, wall chest stretching, and rhomboid stretching. The testing procedure lasted approximately 2 h.

### Drop-Jump Test

After equipment calibration, the participants stood on a 45 cm box with feet shoulder-width apart and moved the body forward slowly and smoothly to drop with no horizontal velocity (Bates et al., 2013). A maximum vertical jump with arm-swing was performed immediately after landing on the two force plates, and each foot contacted a different plate. The participants were instructed to jump as if jumping for a basketball rebound. The impact phase of the landing occurred on two force plates was required for a successful trial. Three successful trials were recorded for each participant, and sufficient time (at least 30 s, longer if the participant requested) was given between each trial to ensure that maximal effort could be exerted.

### Instrumentation and Data Reduction

**FIGURE 1:**
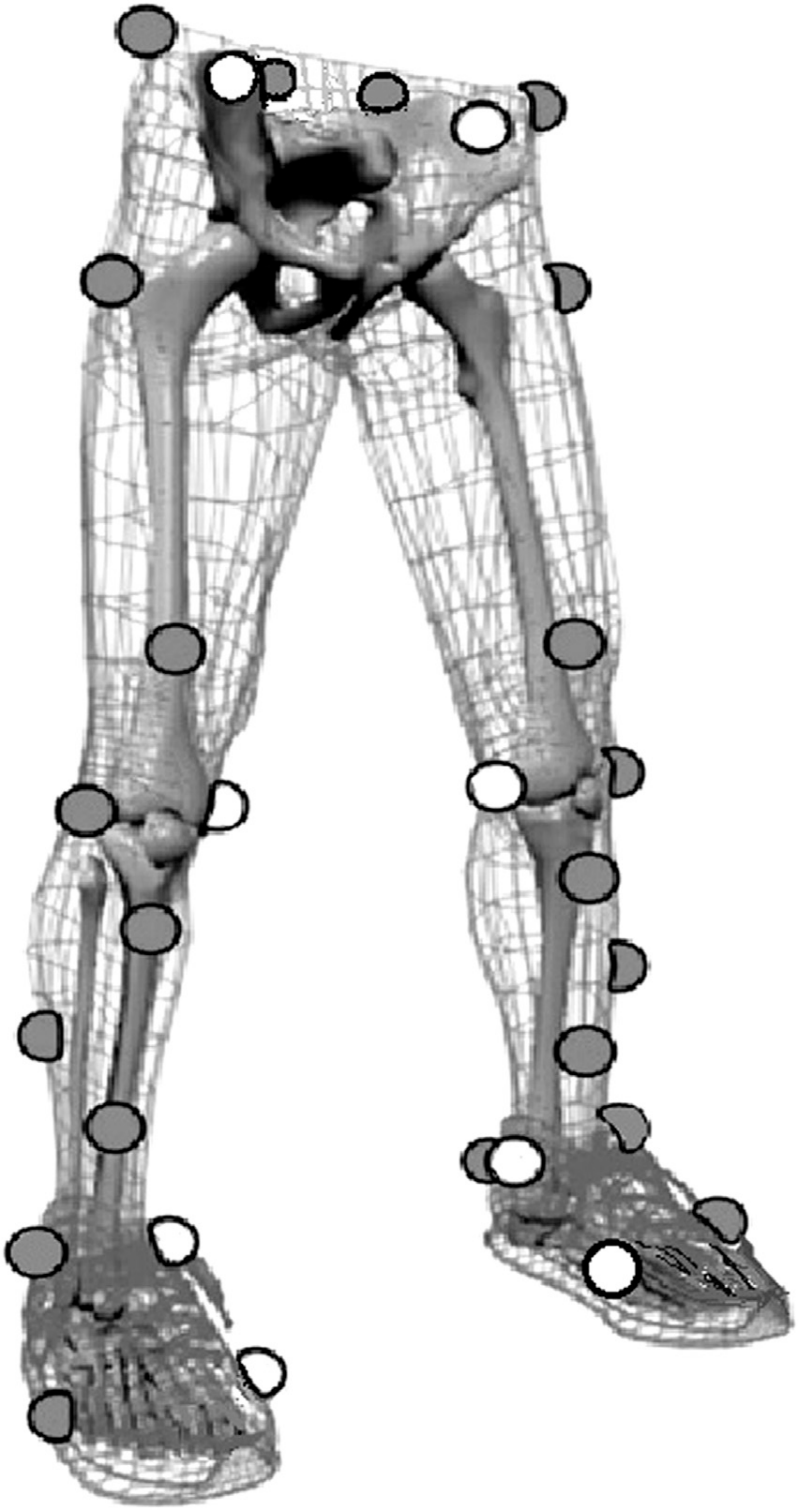
Marker locations used to define the hip, knee, and ankle joints. The white markers, including the left and right anterior superior iliac spine, bilateral medial femoral condyle, bilateral medial malleoli, and bilateral fifth metatarsal heads markers, were removed after static capturing.

Participants were asked to wear the same shoes to eliminate bias caused by wearing different shoes during the DJ test. A Vicon motion capture system (Vicon Metrics Ltd., Oxford, United Kingdom) with eight infrared cameras was used to record the motion during the DJ test, and the sampling frequency was set at 200 Hz. A total of 30 retro-reflective markers (14.0 mm diameter) were attached to the lower-extremity to define the hip, knee, and ankle joints (McLean et al., 2010). Two additional tracking markers around the knee were used to minimize the error of soft tissue artifacts (Leardini et al., 2005). To minimize the error due to anatomical landmarks, one expert researcher who majored in biomechanics was employed to place the markers for all the participants (McGinley et al., 2009). The participants were instructed to stand still on two force plates, and a static trial was collected to align the joint coordinate system to the laboratory before data were collected. Participant’s standing positions were used to implement this alignment and control for interparticipant variation in anatomical alignment (i.e., zero-position abduction alignment) during the static trial. Eight markers (white markers in Figure 1) were removed after static capturing. Two 90 × 60 cm force plates (AMTI, Watertown, Ma, United States) sampled at 1,000 Hz were used to measure the ground reaction forces (GRFs). The force plate system was synchronized with that of the Vicon motion system so that every fifth force data sample occurred at a single corresponding video frame. A plug-in in Vicon motion system provided by the Vicon company was used for synchronization. Static and dynamic calibrations for the force plates and Vicon motion system were conducted according to the instruction (Guo et al., 2018a and Guo et al., 2020).

Visual3D software (C-Motion Inc., Germantown, MD, United States) was used for data reduction and analysis. The force and kinematic data were filtered using a low-pass, zero-lag Butterworth filter with a cutoff frequencies of 44 and 14 Hz, respectively (Guo et al., 2018a and Guo et al., 2020). The landing phase of the dominant leg was analyzed and defined as the time interval from initial contact with the force plate, where the vertical GRF first exceeded 10 N, to maximum knee flexion during the first landing (Bates et al., 2013). The Dominant leg was defined as the leg used to kick a ball. The peak moment, the angle at initial contact, and the maximum angular motion of knee extension, abduction, and internal rotation of the dominant leg were employed to represent the knee injury risk (Cowley, 2006; Bates et al., 2013). All variables were averaged over the three trials. The knee moments were reported as external joint moments and calculated using inverse dynamics from the force and kinematic data. The knee angles and angular motions were calculated with 0° representing the subject’s static standing position.

### Core-related Measurements Test

**FIGURE 2:**
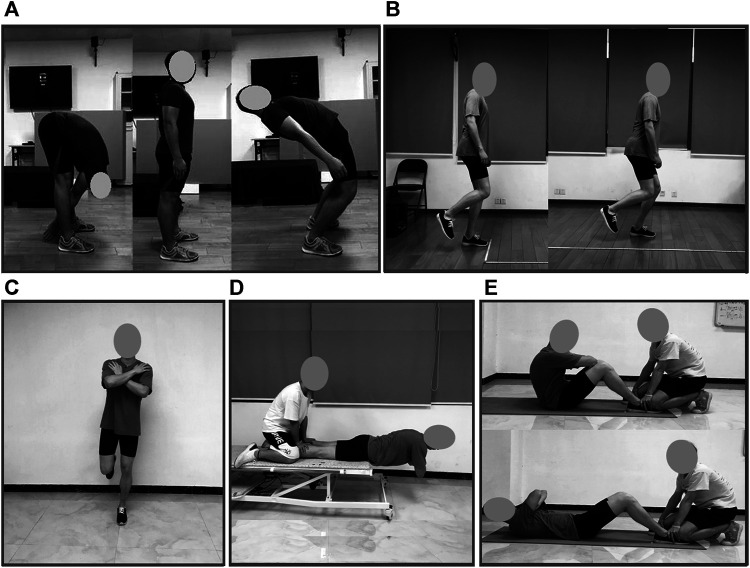
Five core-related measurements. **(A)**. From left to right: trunk flexion (TF), neutral, and extension (TE) range of motion test, where trunk flexion to extension range of motion (TFE) was recorded as the sum of TF and TE. **(B)**. Dominant limb single-leg hop distance (DLH) test. **(C)**. Dominant limb single-leg stance (DLS) test. **(D)**. Trunk extensor endurance (EE) test, and **(E)**. Sit-ups in 20 s (SU) test.

The core-related measurements were obtained via five tests based on the previous research (Waldhelm, 2012; Guo et al., 2018a; Guo et al., 2019): total trunk flexion and extension range of motion (TFE) for the flexibility of the core; dominant extremity single-leg stance time (DLS) test for the motor control of the core; dominant extremity single-leg hop distance (DLH) test for the function of the core; trunk extensor endurance (EE) and sit-ups in 20 s tests for the endurance and strength of the core. Good to excellent intra-rater reliabilities (Intraclass correlation coefficient ≥0.62) of the five tests have been established in the Waldhelm et al (2012) study. See Figure 2.

#### Trunk Flexion and Extension Range of Motion Test

Measurements were taken by measuring the distance between cervical vertebrae 7 (C7) and sacral vertebrae 1 (S1) while standing in a neutral position with the shoes off. These landmarks (C7 and S1) were identified and marked with a pen. Subsequently, participants flexed forward as far as possible while stabilizing their pelvis with the investigator’s instruction, “hunch forward as far as you can, keeping your hips still.” The distance between C7 and S1 was remeasured. The difference between the neutral and flexed positions was recorded as the trunk flexion range of motion (TF). The same protocol was used in evaluating trunk extension, with participants extending back as far as possible and the distance remeasured. The difference between the neutral and extended positions was recorded as the trunk extension range of motion (TE). TFE was recorded as the sum of TF and TE. All parameters were recorded in centimeters, rounded to the nearest tenth of a centimeter by the same experimenter. See Figure 2A.

#### Dominant Extremity Single-Leg Hop Distance Test

Participants performed this test by hopping and landing on their dominant extremity while maintaining their landing for at least 2 s in a successful trial (Figure 2B). The distance hopped was marked with tape and measured from toe to toe. Three hops were performed, with the longest hop recorded in centimeters rounded to the nearest tenth of a centimeter.

#### Dominant Extremity Single-Leg Stance Test

Participants stood on their dominant extremity and raised the other extremity with the knee flexed to 90° with the hips straight and not touching the stance extremity. Participants were instructed to cross their arms over the chest prior to lifting their extremities. This test was performed three times/trials with eyes closed, and the best of the three trials was recorded. Time began when participants raised their non-dominant feet off of the floor. Time was discontinued when they either: 1) uncrossed their arms, 2) touched the floor with their raised foot, 3) moved the foot on the ground to maintain balance, 4) a maximum of 45 s passed, or 5) opened their eyes. Time was recorded in seconds. See Figure 2C for more details.

#### Trunk Extensor Endurance Test

This test was performed with the participant lying prone on a medical bed. Participants kept their right and left anterior superior iliac spines on the edge of the bed to support their body weight with their pelvis, hips, and knees secured by an experimenter. When instructed, participants assumed a horizontal position with arms across their chest, hands on the opposite shoulder, and elbows were pointing vertically down to the floor (Figure 2D). This position was held as long as possible. The primary investigator gave verbal cues every 10 s along with a brief motivational statement during the test. The test was discontinued when they fell below the horizontal position or when their elbows touched the floor. Time was recorded in seconds.

#### Sit-Ups in 20 s Test

This test was initiated in the hook-lying position, with the knees flexed to 90°, arms across the chest with each hand on the opposite shoulder, and the feet secured on the floor by a partner. For a full sit-up to count, participants had to have their scapulae touching the mat in the lying position and the elbows contacting the knees in the sitting position (Figure 2E). The number of repetitions was recorded. Participants performed as many full sit-ups as possible within 20 s. The primary investigator provided verbal cues every 5 s. The cue was a brief motivational statement.

### Data Analysis

All results were analyzed with SPSS for Windows (SPSS version 23.0, IBM Corporation, Armonk, NY, United States). Normality was checked using the Kolmogorov–Smirnov test. A log transformation would be used if the normality was not met and would be rechecked after the transformation. The square of the Pearson correlation coefficient (*R* ^2^) was employed to estimate the shared variance between knee injury risk factors in the DJ test and core-related measurements. A stepwise multiple linear regression was conducted to determine which independent core-related measurements were major predictors, with a significant contribution to the knee injury risk factors obtained in the DJ test. The knee injury risk factors obtained in the DJ test were entered as dependent variables, and the core-related measurements were entered as independent variables. The use probabilities of F were set as 0.05 and 0.10 for entering and deleting, respectively. The significance level was set at *p* ≤ 0.05 for all tests.

Sample size estimation was performed on G*Power software (Germany). The effect size was calculated with the minimum value of the coefficient of determination (*R* ^2^), which is significant in the present study. By setting the level of significance to 0.05 and the statistical power to 0.80 in a two-tailed test on correlation (Abt et al., 2020), the effect size and estimated required sample size were calculated to be 0.47 and 15, respectively. To confirm the power of the significance, a post-hoc power analysis on the correlation between core-related measurements and knee kinetic and kinematic measurements was conducted with G*Power software.

## Results

### Descriptive Statistics for Basic Information, Core-related Measurements, and Knee Injury Risk Factors

Eighteen active college-aged male amateur basketball players (age: 21.9 ± 0.5 years old, body mass: 65.6 ± 1.6 kg, height: 1.74 ± 0.02 m) were enrolled for the study.

Table 2 presents the results of the core-related measurements and knee injury risk factors during the first landing as the mean and standard error of the mean (S.E).

**TABLE 2:** Core-related measurements and kinematic and kinetic measurements during the DJ landing

	Mean	S.E.
DLH/H	1.16	0.02
SU (#)	18.6	0.51
DLS (s)	36.4	1.73
TFE (cm)	18.2	0.79
EE (s)	102.8	6.86
PMKE (Nm/kg)	2.033	0.111
PMKA (Nm/kg)	0.197	0.019
PMKR (Nm/kg)	0.041	0.006
AMKF (°)	109.5	3.4
AMKA (°)	6.6	0.6
AMKR (°)	5.8	0.4
AKFI (°)	36.7	2.7
AKAI (°)	2.5	0.4
AKRI (°)	3.8	0.6

DLH/H, Dominant extremity single-leg hop distance normalized by body height; SU, sit-ups in 20 s; DLS, Dominant extremity single-leg stance time; TFE, Trunk flexion and extension range of motion; EE, Trunk extensor endurance test.

PMKE, Peak moment of knee extension; PMKA, Peak moment of knee abduction; PMKR, Peak moment of knee internal rotation; AMKF, Angular motion of knee flexion; AMKA, Angular motion of knee abduction; AMKR, Angular motion of knee internal rotation; AKFI, Angle of knee flexion at initial contact; AKAI, Angle of knee abduction at initial contact; AKRI, Angle of knee internal rotation at initial contact.

### Correlation Analyses Between Core-related Measurements With Knee Injury Risk Factors

The normality of the variables was confirmed before the correlation analysis. Table 3 shows the shared variance (*R* ^2^) between core-related measurements and knee injury risk factors during landing. Sit-ups in 20 s (SU) has shared significant variance with the peak moment of knee extension (PMKE, *p* < 0.05), the peak moment of knee abduction (PMKA, *p* < 0.05), and angle of knee internal rotation at initial contact (AKRI, *p* < 0.05). Dominant extremity single-leg stance time (DLS) has shared significant variance with the angular motion of the knee internal rotation (AMKR, *p* < 0.05) and AKRI (*p* < 0.01). Table 4 shows the *post-hoc* power analysis on the correlation between core-related measurements and knee kinetic and kinematic measurements. All the power values of the correlation were greater than 0.8.

**TABLE 3:** Shared variance (*R* ^2^) between core-related measurements with kinematic and kinetic measurements during the DJ landing

	PMKE	PMKA	PMKR	AMKF	AMKA	AMKR	AKFI	AKAI	AKRI
DLH/H	0.159	0.002	0.043	0.059	0.000	0.054	0.082	0.003	0.061
SU	0.281^a^	0.222^a^	0.070	0.002	0.013	0.054	0.001	0.189	0.347^a^
DLS	0.186	0.040	0.049	0.110	0.010	0.279^a^	0.027	0.016	0.366^b^
TFE	0.069	0.063	0.037	0.100	0.011	0.042	0.009	0.000	0.099
EE	0.021	0.040	0.003	0.001	0.001	0.011	0.070	0.000	0.011

a *p* < 0.05.

b *p* < 0.01.

**TABLE 4:** *Post-hoc* power analysis on the correlation between Core-related measurements and knee kinetic and kinematic measurements

	Effect size	Power
SU-PMKE	0.53	0.95
SU-PMKA	0.47	0.88
SU-AKRI	0.59	0.99
DLS-AMKR	0.53	0.95
DLS-AKRI	0.61	0.99

### Linear Regression Analyses for Knee Injury Risk Factors and Core-Related Measurements

**FIGURE 3:**
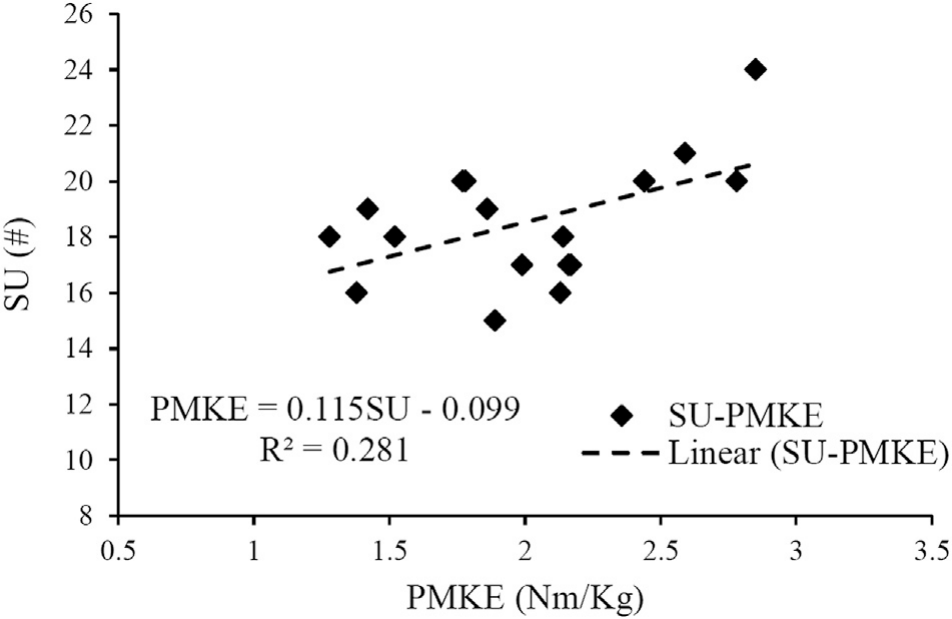
Results of linear regression analyses of the peak moment of knee extension (PMKE) with the five core-related measurements, which included dominant extremity single-leg hop distance normalized by body height (DLH/H), sit-ups in 20 s (SU), dominant extremity single-leg stance time (DLS), trunk flexion and extension range of motion (TFE), trunk extensor endurance (EE).

Figure 3 shows the linear regression analyses for PMKE and the core-related measurements. The regression model demonstrated that SU was the only variable left in the regression analysis, and it explained 28% of the variance observed in the PMKE (*p* < 0.05). The possibility of collinearity was low since the variance inflation factor (VIF) was at 1.000.

**FIGURE 4:**
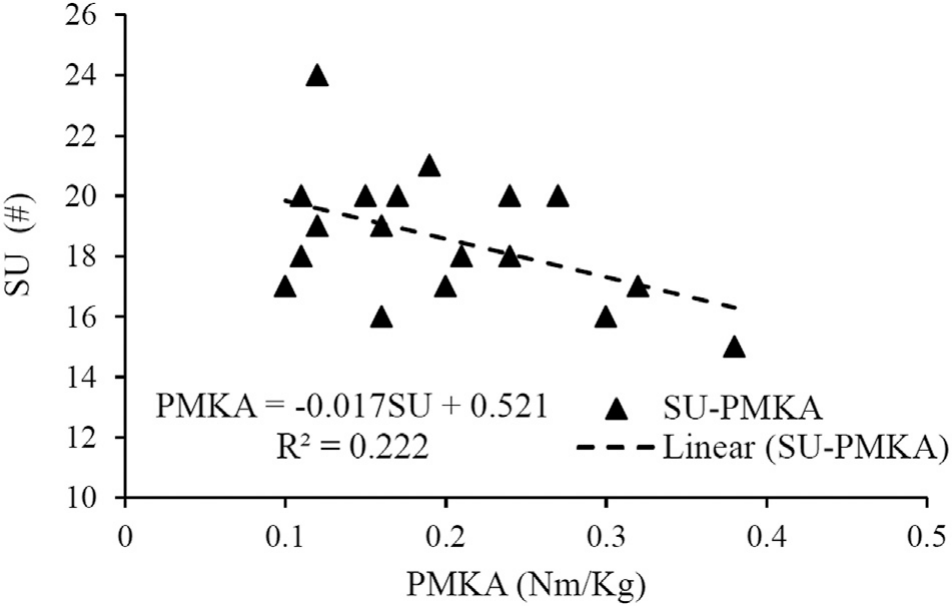
Results of linear regression analyses of the peak moment of knee abduction (PMKA) with the five core-related measurements, which included dominant extremity single-leg hop distance normalized by body height (DLH/H), sit-ups in 20 s (SU), dominant extremity single-leg stance time (DLS), trunk flexion and extension range of motion (TFE), trunk extensor endurance (EE).

Figure 4 shows the results of the linear regression analyses of the PMKA and core-related measurements. SU was the only variable left in the regression analysis, and it explained 22% of the variance observed in the PMKA (*p* < 0.05, VIF = 1.000).

**FIGURE 5:**
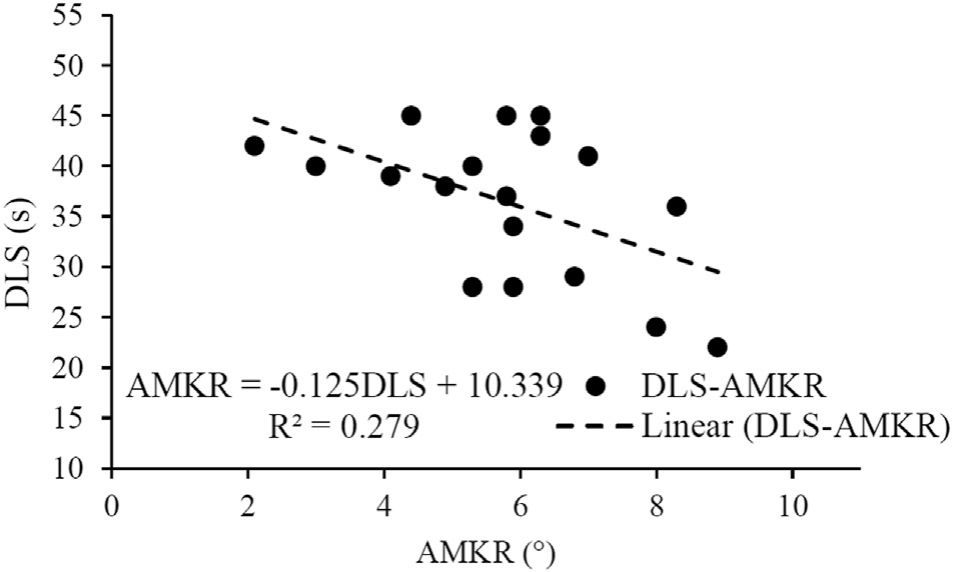
Results of linear regression analyses of the angular motion of the knee internal rotation (AMKR) with the five core-related measurements which included dominant extremity single-leg hop distance normalized by body height (DLH/H), sit-ups in 20 s (SU), dominant extremity single-leg stance time (DLS), trunk flexion and extension range of motion (TFE), and trunk extensor endurance (EE).

Figure 5 shows the linear regression analyses of the AMKR and core-related measurements. DLS was the only variable left in the regression analysis, and it explained 22% of the variance observed in the PMKA (*p* < 0.05, VIF = 1.000).

**FIGURE 6:**
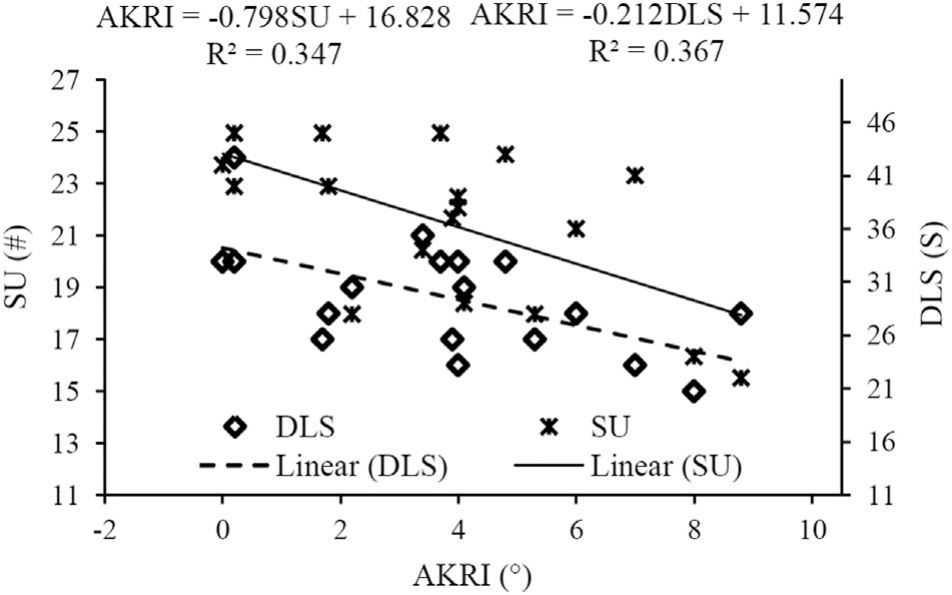
Results of linear regression analyses of the knee internal rotation angle at initial contact (AKRI) with the five core-related measurements, which included dominant extremity single-leg hop distance normalized by body height (DLH/H), sit-ups in 20 s (SU), dominant extremity single-leg stance time (DLS), trunk flexion and extension range of motion (TFE), and trunk extensor endurance (EE).

Figure 6 shows the linear regression analyses of the AKRI and core-related measurements. The regression model demonstrated that variation in SU and DLS explained 52% of the variation in the AKRI (*p* < 0.05), and the equation was AKRI = 18.829–0.157DLS-0.499SU. The possibility of collinearity was low since the VIF for both independent variables was at 1.165.

## Discussion

We set out to study the relationship between five core-related measurements and knee injury risk during DJ landing. The results indicated that sit-ups in 20 s (SU) shared significant variance with the PMKE, PMKA, and AKRI. Dominant extremity single-leg stance time (DLS) shared significant variance with the AMKR and AKRI. SU and DLS could significantly predict 52% of the variance in the AKRI. Core strength and motor control are two specific aspects of the core stability to prevent knee injury during DJ landing, and different aspects played different roles.

The knee kinematic variables, especially for the angles of abduction and internal rotation in the present study, were collected in potential error due to anatomical landmarks and soft tissue artifacts (Della Croce et al., 1999; Leardini et al., 2005). We have tried our best to minimize the error all through the experiment process, e.g. more tracking markers around the knee and one expert researcher placing the markers for all the participants and so on (Leardini et al., 2005; McGinley et al., 2009). The coefficient of variation (CV) of AMKA, AMKR, AKAI, and AKRI were 39, 29, 60, and 68%, respectively, which were lower than previous researches (67, 46, 1017, 135% in the research of Tsai et al. (2019) and 143, 94, 83, and 121% in the research of Everard et al. (2019). The decreased CV of AMKA, AMKR, AKAI, and AKRI compared with those of previous research (Everard et al., 2019; Tsai et al., 2019) could partly demonstrate a promotion of the accuracy for the present study. However, we still need to be cautious when discussing the significant correlations with the AKRI and AMKR in the present study.

Excessive knee abduction loading during landing is directly related to knee injuries (Hewett et al., 2005; Stuelcken et al., 2016). Hewett et al. (Hewett et al., 2005) prospectively demonstrated that the peak knee abduction moment during landing predicted ACL injury risk with 78% sensitivity and 73% specificity. In addition, individuals who sustained an ACL injury displayed peak knee abduction moments during landing that were 2.5 times greater on average than the corresponding values in uninjured individuals (Hewett et al., 2005). Core stability is essential in keeping the body stable and preventing lower-extremity injury during landing (Leetun et al., 2004; Myer et al., 2008; Araujo et al., 2015). A stable core could reduce the overturning and rotating torque on the lower-extremity joints when landing and effectively alleviate the relevant joints loading (Zazulak et al., 2007a; Bagherian, 2018). An inadequate core may compromise dynamic stability of the knee and result in increased abduction moment, which may increase strain on the knee soft tissues and lead to injury (Zazulak et al., 2007a). In the present study, SU correlated with the PMKE significantly (*p* < 0.05) and negatively correlated with the PMKA significantly (*p* < 0.05), highlighting the important role of the core in buffering the ground impact force and alleviating knee loading. Core strength could make the trunk rigid as a cylinder (Hibbs, 2008; Guo et al., 2020), strong and stable, which helps keep the whole body stable and decrease the disturbing force on the knee during landing. The PMKA certainly correlated with SU significantly. The PMKE helps buffer the ground impact force during landing. The significant correlation between SU and PMKE in the present study demonstrated the critical role of core strength in helping the knee absorbing energy during landing.

In previous studies investigating the relationship between core stability and lower-extremity injury, integrative training included various approaches to addressing core stability (DiStefano et al., 2016; Tsai et al., 2019). More than two aspects were chosen from strength, endurance, flexibility, motor control, function and etc of core stability in the integrative training. Different aspects of core stability play different roles in preventing lower-extremity injury. It is difficult to determine which training elements are responsible for preventing knee injury during landing (Parsons et al., 2017; Guo et al., 2020). The concept of five categories of core stability based on previous research (Waldhelm, 2012; Guo et al., 2018a; Guo et al., 2019) was employed in the present study to determine the specific relationship between knee injury risk and core stability. Waldhelm and Li (2012) identified 35 core-related measurements from previous studies and classified them into five categories by principal component analysis. The five categories were strength, endurance, flexibility, motor control, and function of the core. Based on Waldhelm’s research, Guo et al. (2018a) and Guo et al. (2018b) screened five measurements, which were sit-ups in 20 s (SU), trunk extensor endurance (EE), trunk flexion and extension range of motion (TFE), dominant extremity single-leg stance time (DLS), and dominant extremity single-leg hop distance (DLH), to represent the five categories of core stability respectively and disclose the “specificity” of core stability to predict countermovement jump performance. However, no study has been devoted to the relationship between the five categories of core stability and landing performance. In the present study, we also employed the five measurements base on the previous research (Waldhelm, 2012; Guo et al., 2018a; Guo et al., 2020). We investigated the relationship between core stability and knee biomechanics during landing. The results indicated that SU shared significant variance with the PMKE, PMKA, and AKRI. DLS shared significant variance with the AMKR and AKRI. The results revealed that the different components of core stability played different roles in knee injury risk during landing and supported our hypothesis.

The 3-dimensional angles of the knee at initial contact and maximal displacement when landing are crucial factors affecting knee injuries, especially ACL injuries (Della Villa et al., 2020; Xu et al., 2020). Previous research has found that subjects undertaking a DJ task with less knee flexion and greater knee internal rotation and knee abduction at initial contact and maximal displacement were associated with a greater rate of knee injury in sports (Dai et al., 2012; Everard, 2019). In the present study, SU and DLS were negatively correlated with the AKRI and shared a 52% variance. In the present study, the core strength represented by SU could stabilize the core and correct posture and movement patterns during landing (Araujo et al., 2015). After core control and strength training, a decreased knee internal rotation angle during landing was also observed in Tsai et al. (2019) study. DLS with eyes closed represented core motor control in the present study and was highly dependent on proprioception, which is the ability to integrate sensory information to maintain awareness of the positions of the body’s segments and joints (Han et al., 2016). It is crucial to control the knee motion and prevent knee injury, and DLS was also negatively correlated with the AMKR significantly in the present study. Individuals who have good proprioception could activate lower-extremity muscles earlier and promote the function of the lower-extremity muscles during landing (Zazulak et al., 2007b; Han et al., 2016), which may explain the negative correlation between DLS with AKRI and AMKR. Angular motion during landing could affect knee loading and injury risk (Cowley, 2006; Vander Does et al., 2016). Excessive angular motion increases strain on the knee’s muscle, cartilage, ligaments and lead to injury (Tsai et al., 2019). Thus, the negative correlations between DLS with AKRI and AMKR implied the importance of core motor control in preventing knee injury.

We selected five core-related measurements in the present study and investigated their relationship with knee kinematic and kinetic measurements during landing. We found the specific measurements of the core in preventing knee injury. However, we only had 18 amateur basketball players, and the statistical significance was not very high in the present study. A greater number and other types (e.g., volleyball and other professional athletes) of participants should be further investigated. Also, the observed correlations were of a magnitude outside of the protocol’s measurement error. The potential error due to anatomical landmarks and soft tissue artifacts should be considered when discussing the kinematic data of the knee, especially for the angles of abduction and internal rotation in the present study. In addition, the participants conducted an anticipated DJ in the laboratory, which is different from knee injuries in the fields. Thus the results could only reflect knee injury within certain limits.

## Conclusion

Our results showed that sit-ups in 20 s (SU) shared significant variance with amateur basketball player’s PMKE, PMKA, and AKRI. Dominant extremity single-leg stance time (DLS) shared significant variance with the AMKR and AKRI. Core strength and motor control were two specific aspects of core stability related to preventing knee injury during DJ landing, and different aspects played different roles. An integrative training program addressing varied and specific aspects of core stability could be considered for basketball, and other sports, coaches and athletes to prevent knee injury through core training and conditioning.

## Data Availability

The original contributions presented in the study are included in the article, further inquiries can be directed to the corresponding author.
